# Osteopontin is elevated in patients with mitral annulus calcification independent from classic cardiovascular risk factors

**DOI:** 10.1186/s12872-016-0314-3

**Published:** 2016-06-10

**Authors:** Michael Sponder, Christian Reuter, Monika Fritzer-Szekeres, Brigitte Litschauer, Thomas Binder, Jeanette Strametz-Juranek

**Affiliations:** Department of Cardiology, Medical University of Vienna, Währinger Gürtel 18-20, 1090 Vienna, Austria; Department of Anesthesiology and Operative Intensive Care Medicine, University Hospital of Bonn, Sigmund-Freud-Straße 25, 53127 Bonn, Germany; Department of Medical-Chemical Laboratory Analysis, Medical University of Vienna, Währinger Gürtel 18-20, 1090 Vienna, Austria; Department of Clinical Pharmacology, Medical University of Vienna, Währinger Gürtel 18-20, 1090 Vienna, Austria

**Keywords:** Osteopontin, Mitral annulus calcification, Coronary artery disease, Atherosclerosis

## Abstract

**Background:**

Osteopontin (OPN) regulates the Ca^++^-deposition in bone and coronary arteries. Elevated OPN were also associated with (aortic) valve calcification in healthy individuals. This study aimed to investigate the association between OPN levels and mitral annulus calcification (MAC) in patients with coronary artery disease (CAD).

**Methods:**

In this cross-sectional study OPN-levels were measured in 223 non-or ex-smoking patients (160 male, mean age: 61,09 ± 11,02 years; 63 female: mean age: 67,49 ± 7,87 years) with CAD. Plasma OPN levels were measured by ELISA and MAC was evaluated by echocardiography.

**Results:**

Forward stepwise logistic regression analysis (likelihood quotient) showed significantly higher OPN-levels in patients with MAC compared to patient without calcified mitral annulus independent from the classic risk factors age and severity of coronary artery disease (CAD). In addition to age and the severity of CAD, the circulating OPN amount was a significant predictor for MAC.

**Conclusions:**

This is the first clinical trial which observed increased circulating OPN levels in MAC, suggesting a distinct role of OPN in the process of MAC. Considering the current knowledge about OPN it is more likely that OPN does not promote but counteracts valve calcification and therefore is elevated in course of a calcification processes.

## Background

MAC (the calcification of the mitral valve supporting ring) is a chronic degenerative process and it is therefore not surprising that the prevalence of MAC in patients suffering severe CAD is significantly higher compared to patients without CAD (15 % [1] vs. 35 % [2]). Results from the Framingham Heart study showed that MAC predicts incident cardiovascular events and all-cause death [3]. Furthermore, complete posterior mitral valve annular decalcification with MV repair of replacement represents a serious risk in mitral valve surgery [4].

OPN, an acidic phosphorylated glycoprotein, was suggested as a kind of “survival factor” for different types of cells [5] and has angiogenic potential due to activation of PI3K (phosphoinositide 3-kinase)/AKT (protein kinase B)- and ERK (extracellular signal-kinase) pathways through VEGF (vascular endothelial growth factor) in endothelial cells [6]. It is closely associated with calcified deposits that were found in atherosclerotic lesions, kidney stones and also tumors [7]. It was shown to be absent in native non-calcified human aortic valves but present in minimal and highly calcified ones [8]. Similar results were obtained for rheumatic and non-rheumatic mitral valves [9, 10]. A correlation of elevated plasma levels of OPN and AVC (aortic valve calcification) was also found in healthy elderly subjects [11] and patients suffering CAD [12]. On the one hand OPN is involved in the process of calcification in bones [13] but on the other hand it was also shown to stimulate bone resorption [14]. Concerning vascular calcification, Wada et al. showed in a cell culture system that exogenous OPN potently inhibited calcification by inhibition of apatite growth [15].

OPN doubtlessly plays an important role in CAD but at present the role of its circulating amounts in MAC is not clear. Consequently, the present study investigated whether circulating plasma OPN amounts are elevated in patients with MAC and CAD.

OPN doubtlessly plays an important role in CAD but at present the role of its circulating amounts in MAC is not clear. Consequently, the present study investigated whether circulating plasma OPN amounts are elevated in patients with MAC and CAD. The further investigation of the role of OPN in the process of valve but also vessel calcification might be important when thinking about possible medical therapies.

## Methods

### Study population

In total 160 male (mean age: 61,09 ± 11,02 years) and 63 female (mean age: 67,49 ± 7,87 years) consecutive patients, never-smoking or ex-smoking for at least 7 years, with angiographycally verified CAD of different severity were recruited. All patients underwent a coronary angiography for diagnostic and/or therapeutic reasons on grounds of their underlying disease. The coronary artery system was divided into 17 segments and stenosis grade for each segment was measured. A simple 3-point-grading system (“Coronary Score” [16]) was developed considering both frequency and severity of CAD. The patients received 0 points for non-stenosed or only calcified segments, 1 point for each stenosis from <30- < 50 %, 2 points for each stenosis from 50- < 70 % and 3 points for each stenosis >70 %. As the coronary score represents the total coronary artery calcification grade it is indirectly also a surrogate for the influence of cardiovascular risk factors in the coronary artery system.

The study protocol has been approved by the Ethics Committee of the Medical University of Vienna. The work has been carried out in accordance with the Declaration of Helsinki; written informed consent was obtained from all subjects.

### Echocardiographic investigation

Echocardiographic data were obtained with the use of commercially available ultrasound systems (GE Medical Systems Vivid 7 Dimensions, Horton, Norway). Echocardiography was performed without knowing OPN-levels and was therefore “blinded”. MAC was assessed in an apical long axis and parasternal short axis view. Patients with surgical interventions of the mitral valve and/or mitral stenosis were excluded for the evaluation of MAC because they are associated with valvular calcification processes per se. MAC was defined by echocardiography as a dense, highly reflective area at the base of the mitral-valve leaflets [17] located at the junction of the atrioventricular groove and the posterior or anterior mitral leaflet seen on the parasternal long-axis or short axis view or on the apical 4- or 2-chamber view. The severity of MAC, expressed as maximal thickness in millimetres, was measured from the leading anterior to the trailing posterior edge at its greatest width. Calcification thickness >1 mm and <4 mm was considered mild to moderate, and >4 mm was considered severe [18].

### Laboratory analysis

Routine laboratory analysis was conducted by the Department of Medical-Chemical Laboratory Analysis of the Medical University of Vienna. The full-length form of OPN was analysed in plasma by Enzyme-linked Immunosorbent Assay (ELISA) according to the instructions of the manufacturer (R&D Systems, Minneapolis; sensitivity: 0,063 ng/ml; assay range: 0,310-10 ng/ml). The coefficient variation (CV) was about 7 %.

### Statistical analysis

Statistical analysis was done with SPSS 20.0. Continuous and normally distributed data is described by means ± standard deviation (SD) and group differences were tested by independent sample *t*-test or ANOVA. Not normally distributed variables are presented as median and minimum/maximum and Mann–Whitney *U*-test or Kruska-Wallis H-test were applied to test for group differences. To determine whether a significant influence of OPN-concentration on MAC persisted after controlling for other variables a forward stepwise logistic regression analysis (likelihood quotient) was performed with the following covariates: age, sex, coronary score, diabetes and diastolic blood pressure. These covariates were chosen because first, they present classic risk factors and second, univariate analysis showed a significant difference in these covariates in patients with and without MAC.

All tests were performed two-sided and *p*-values ≤ 0,05 were considered significant.

## Results

### Description of patients

Important anthropometric data, blood pressure, laboratory routine parameters, prevalence of the main cardiovascular risk factors and the severity of CAD (Coronary score) are shown in Table 1. Patients with MAC were about 10 years older (*p* <0,001) and had higher creatinine (1,06 vs. 1,30 mg/dl; *p* = 0,008) and proBNP levels (1372 vs. 3655 ng/ml; *p* = 0,001) and they suffered more often diabetes mellitus (20,7 vs. 43,0 %; *p* <0,01). However, in MAC-patients diastolic blood pressure was significantly lower (73 vs. 78 mmHg; p = 0,004) and HDL-cholesterol levels were significantly higher (44 vs. 48 mg/dl; *p* = 0,04).

**Table 1:** Patient description: Anthropometric data, systolic and diastolic blood pressure (SBP, DBP), heart rate (HR), medication, prevalence of CV-risk factors and the severity of CAD represented by the coronary score. Data is given as mean ± SD or %/n; *marks significant differences between the both groups

Parameter	No MAC (*n* = 144)	MAC (*n* = 79)	*p*-value
Age (years)	59,48 ± 10,41	69,15 ± 7,79	<0,01*
BMI (kg/m^2^)	28,51 ± 5,19	27,52 ± 4,93	0,160
SBP (mmHg	129,69 ± 15,63	131,70 ± 17,76	0,387
DBP (mmHg)	77,77 ± 11,03	73,18 ± 11,10	0,004*
HR (bpm)	70,56 ± 12,67	70,51 ± 14,26	0,976
Na (mmol/l)	138,72 ± 2,60	139,24 ± 3,09	0,184
K (mmol/l)	4,06 ± 0,48	4,09 ± 0,42	0,607
Cl (mmol/l)	103,78 ± 4,01	104,11 ± 3,30	0,532
Uric acid (mg/dl)	6,58 ± 2,13	6,99 ± 2,15	0,182
Creatinine (mg/dl)	1,06 ± 0,40	1,30 ± 0,93	0,008*
BUN (mg/dl)	18.06 ± 10,53	24,32 ± 13,34	<0,01*
Triglycerides (mg/dl)	163,65 ± 94,13	136,55 ± 60,83	0,023*
Cholesterol (mg/dl)	175,96 ± 51,08	174,31 ± 47,38	0,814
HDL (mg/dl)	43,60 ± 12,22	47,61 ± 14,11	0,040*
LDL (mg/dl	103,21 ± 39,42	104,95 ± 44,45	0,780
HbA_1_C (rel%)	6,13 ± 1,05	6,46 ± 1,20	0,057
Erythrocytes (T/l)	4,59 ± 0,58	4,38 ± 0,61	0,010*
Haemoglobin (mg/dl)	13,51 ± 1,79	12,58 ± 1,77	<0,01*
Haematocrit rel%	40,05 ± 4,67	37,93 ± 5,00	0,002*
Thrombocytes (G/l)	239,87 ± 82,23	235,48 ± 82,96	0,705
Leukocytes (G/l)	7,93 ± 2,74	7,27 ± 2,43	0,200
TSH (mycroU/ml)	2,09 ± 1,70	2,13 ± 1,71	0,876
proBNP (ng/ml)	1372,70 ± 2585,22	3655,18 ± 6918,58	0,001*
Hypertension	92,4	93,7	0,728
Hypercholesterinemia	93,1	87,3	0,149
Diabetes mellitus	20,7	43,0	<0,01*
Adipositas (BMI >30 kg/m^2^)	40,3	44,2	0,100
Beta-blockers	84,0	77,2	0,210
ACE-Inhibitors	59,0	50,6	0,228
Ca^++^-antagonists	9,7	17,7	0,085
Diuretics	29,9	48,1	0,007*
Statins	85,4	77,2	0,124
Coronary score (points)	6,66	8,96	0,003*
Stenosis left main	15,2	16,5	
Stenosis LAD (prox./med./dist.)	51,0/53,1/20,7	55,7/59,5/17,7	
Stenosis CX (prox./med./dist.)	25,5/17,9/13,1	36,7/26,6/12,7	
Stenosis RCA (prox./med./dist.)	42,1/38,6/26,9	57,0/48,1/38,0	

### Severity of coronary artery disease

The coronary score, representing the severity of CAD and indirectly the burden of cardiovascular risk factors on coronary arteries, was significantly higher in patients with compared to patients without MAC (6,66 vs. 8,96 points; *p* = 0,003). The most frequently affected coronary segments were the medial LAD (left anterior descending) and the proximal RCA (right coronary artery) followed by proximal LAD and medial RCA (see Table 1).

### OPN in MAC

**Fig. 1:**
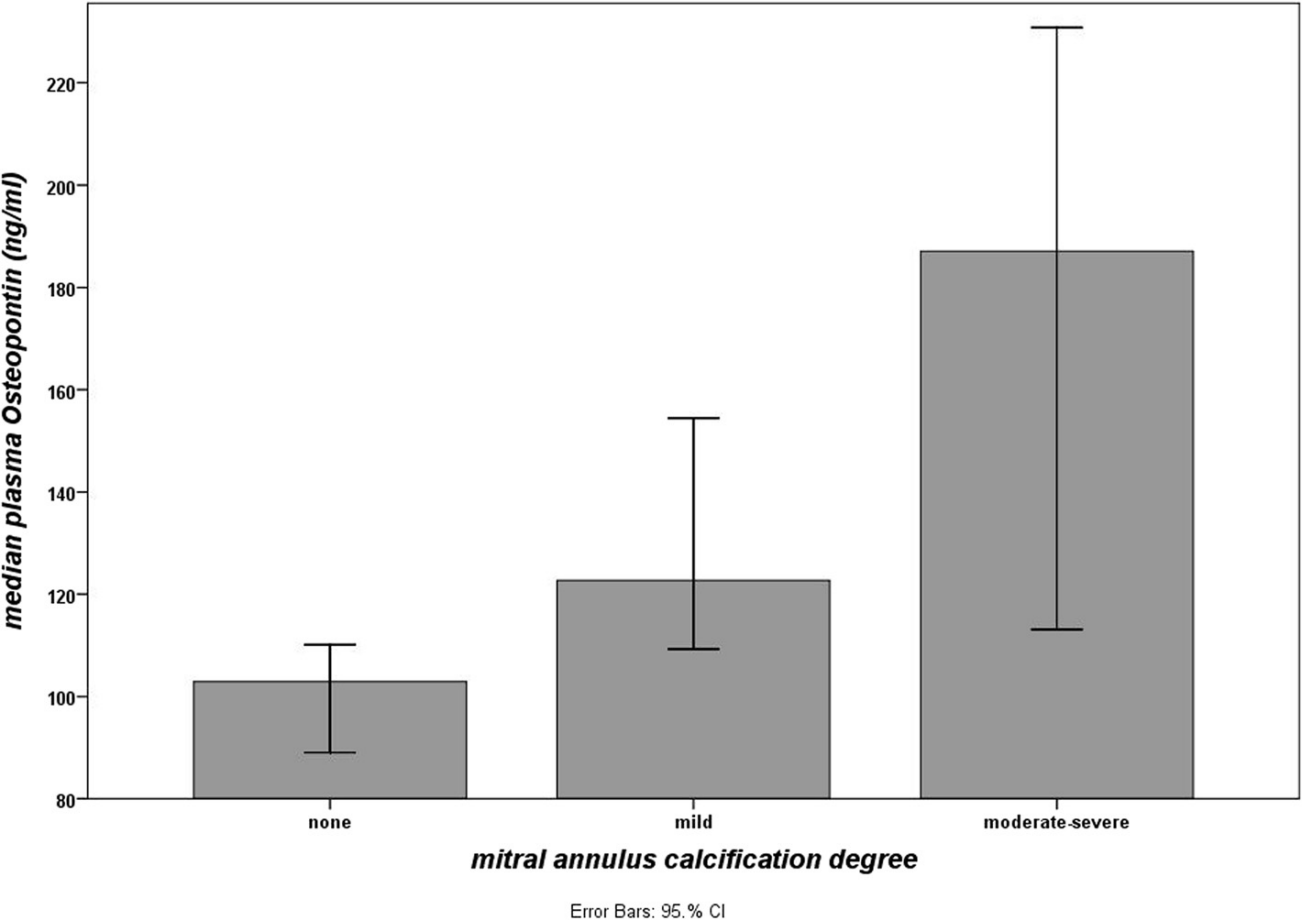
OPG in MAC. Median plasma OPN levels (ng/ml) depending on the degree of mitral annulus calcification

The increase in OPN dependent on the grade of MAC is shown in Fig. 1. Within our population 35,4 % of CAD patients also suffered MAC. We observed a significant increase of OPN levels (*χ*^2^ = 20,3; p <0.001) from 102,9 ng/ml in patients without MAC to 122,7 ng/ml in patients with mild MAC and 187 ng/ml in patients with moderate or severe MAC, corresponding to an increase of 83,3 % (see Table 2). Post-hoc testing showed that the OPN differed significantly between no MAC and each MAC group (*p* = 0,023; *p* = 0,001), but not between the two MAC groups (p = 0,135). Thus, for further analyses both MAC groups were combined. In the logistic regression model the only significant predictors for the likelihood of MAC were age, coronary score and OPN (see Table 3). The model was statistically significant (*χ*^2^ = 52,5, *p* <0.001) and correctly classified 56,8 % of cases.

**Table 2:** OPN in MAC. Plasma OPN levels (ng/ml) in dependence of severity of MAC. Data is given as median/minimum/maximum

	OPN (ng/ml)
No MAC (*n* = 144)	102,9/22,7/480,2
Mild MAC (*n* = 47)	122,7/57,8/389,8
Mod./Sev. MAC (*n* = 32)	187,0/55,2/348,8

**Table 3:** Logistic regression analysis of the MAC status. MAC coded 0 = no MAC, 1 = MAC

	Regression coefficient B	Standard error	Wald	Significance	Odds ratio e^b^
Age (years)	.090	.019	21,533	<0,001	1,094
Coronary score (points)	.068	.031	4,943	0,026	1,071
OPN (ng/ml)	.007	.003	7,443	0,006	1,007
Constant	−7,891	1,329	35,261	<0,001	

*X* ^2^ = 52,5 *p* < 0,001

Nagelkerke *R* ^2^ = 0,312

The exponential function of the regression coefficient (e^b^) is the odds ratio associated with a one-unit increase in the exposure; the given form is correct

## Discussion

The mitral valve is a flow-regulating structure, permanently exposed to (mechanical) sheer stress and therefore the outer layer is a highly stressed structure per se. In case the structural integrity of the outer layer of this valve gets impaired (by numerous noxa) it becomes peculiarly vulnerable. Ectopic calcification meaning the calcification of soft tissue such as (coronary) arteries or heart valves, is an actively regulated process mediated by several proteins. As MAC is a chronic degenerative process the risk factors of MAC basically comprise those of atherosclerosis e.g. age, diabetes, hyperlipdemia, smoking, hypertension, increased BMI, renal insufficiency but also, in contrary to CAD, female sex [19]. Nevertheless, studies have shown that a treatment with ACE inhibitors and statins does not lead to a regression of aortic valve calcification; however, there exist no further studies dealing with a drug treatment against MAC [20]. The role OPN in heart valve calcification and in particular MAC, especially in patients with CAD, is nearly unexplored. In the present study we measured circulating plasma levels OPN in 223 patients with angiographycally verified and quantified CAD whereby 35,4 % of them had MAC.

OPN was shown in former studies to be present in both living aortic valve tissue and calcified areas of bioprosthetic heart valves [21] and in plasma of healthy subjects with aortic valve calcification [11]. A further study by Abdel-Azeez et al. showed that OPN levels not only correlate with MAC and AVC but also that OPN is an independent predictor of MAC and aortic valve sclerosis. Additionally, plasma OPN levels were shown to reflect the extent of coronary stenosis and can be used as a biomarker to identify patients with coronary atherosclerosis [22]. Steitz et al. [23] could show that OPN acts as natural inhibitor of ectopic calcification. In the present study, compared to patients without MAC, patients with mild calcification had about 19,2 % and patients with moderate or severe calcification about 81,7 % higher OPN levels. A stepwise increase in OPN was shown before for aortic valve calcification [24] but not for MAC. Valve calcification is characterized by Ca^++^- deposition and accumulation resulting from several circumstances (e.g. aging and inflammation) [25]. Our results suggest that bone matrix proteins such as OPN, which regulates Ca^++^-deposition in bone and coronary arteries [26], might also be involved in calcification of mitral valves in patients suffering CAD. All of our patients suffered CAD and were homologous concerning e.g. cardiovascular risk factors and routine laboratory parameters but OPN was significantly higher in patients with moderate and severe mitral valve calcification compared to patients without calcifications suggesting a distinct involvement of OPN in valve calcification, despite the presence of CAD. The results of a study by Wada et al. [15] suggested OPN a potent preventive factor of vascular calcification. Thus, it seems also feasible that OPN does not promote but counteracts valve calcification by blocking hydroxyapatite crystal growth and inducing expression of carbonic anhydrase II in monocytic cells and therefore is elevated in course of the calcification processes. A further interesting aspect appears from a recently published study [27] dealing with the influence of physical activity on OPN-levels in CAD-patients. The results of this study showed a linear decrease of circulating OPN-levels in female and male patients with CAD depending on the degree of physical activity. Following the hypothesis that OPN is first a maker for the calcification status and second able to lead to regression of ectopic calcification, our results together with the results of the above mentioned study would emerge the aspect that physical activity might be protective not only by influencing CAD-risk factors but also MAC. However, data from clinical studies (in human) are rare but essential to pursue these hypotheses.

## Conclusion

This is the first clinical trial which observed higher circulating OPN levels in MAC in a large study group of patients with angiographycally verified CAD suggesting a distinct role of OPN in the process of MAC. Considering the current knowledge about OPN it is more likely that OPN does not promote but counteracts valve calcification and therefore is elevated in course of a calcification processes. However, due to the limited knowledge concerning the molecular mechanisms OPN is or might be involved in further studies are needed to investigate the influence of this biomarker in MAC.

## Limitations

Although in total 223 patients suffering CAD were recruited the number of female patients was low as well as the number of patients with mild MAC. A point of criticism might be that OPN is per se strongly influenced by the presence of CAD. However, for that reason we had a broad number of CAD-patients without MAC delivering reliable baseline levels. The single centre nature of the study may limit generalizability.

## Abbreviations

AKT, protein kinase B; BNP, brain natriuretic peptide; CAD, coronary artery disease; CV, coefficient variation; CX, circumflex; ELISA, Enzyme-linked Immunosorbent Assay; ERK, extracellular signal-kinase; HDL, high density lipoprotein; LAD, left anterior descending; LDL, low density lipoprotein; MAC, mitral annulus calcification; OPN, osteopontin; PI3K, phosphoinositide 3-kinase; RCA, right coronary artery; SD, standard deviation; VEGF, vascular endothelial growth factor.
